# Trastuzumab monotherapy for bone marrow metastasis of breast cancer: A case report

**DOI:** 10.3892/ol.2014.1999

**Published:** 2014-03-24

**Authors:** LONG XU, FANG GUO, SHUXI SONG, GUOJING ZHANG, YONGYE LIU, XIAODONG XIE

**Affiliations:** Department of Oncology, PLA Cancer Center, General Hospital of Shenyang Military Region, Shenyang, Liaoning 110840, P.R. China

**Keywords:** bone marrow metastasis, breast cancer, HER-2, trastuzumab, monotherapy

## Abstract

The current study presents the case of a 41-year-old female patient who received modified radical mastectomy and adjuvant chemotherapy and radiotherapy for infiltrating ductal cancer of the left breast. The pathological stage of the disease was IIA. In addition, the patient was negative for the estrogen and progesterone receptors, and human epidermal growth factor receptor-2 gene amplification was identified. At one year following surgery, the patient presented with severe pancytopenia and pain at multiple sites all over the body. Furthermore, the patient’s Eastern Cooperative Oncology Group performance status score was 3 and numeric rating scale pain score was 8. The bone marrow puncture indicated bone marrow metastatic cancer, and the positron emission tomography/computed tomography (CT) indicated multiple internal organ metastases and osseous metastasis. Chemotherapy treatment posed great risks due to the patient’s poor performance status and severe bone marrow suppression. Therefore, trastuzumab monotherapy was administered at a loading dose of 8 mg/kg and a maintenance dose of 6 mg/kg every three weeks. Following four doses of trastuzumab treatment, the patient’s performance status significantly improved and the peripheral blood cell counts had returned to within the normal ranges. Taxol was added to the trastuzumab treatment and seven cycles were completed. No metastatic cancer cells were found in the subsequent bone marrow smear test; however, CT showed metastatic foci in the left lung. Furthermore, the enlarged lymph nodes had subsided and the tumor in the right appendix region had decreased in size by 50%. The patient’s disease condition was maintained stable for 11 months.

## Introduction

Metastatic cancer of the bone marrow develops when malignant tumor cells of non-hematopoietic systems metastasize to the bone marrow by means of hematogenous dissemination or direct invasion. Micrometastasis in the bone marrow is detected in 30–40% of breast cancer patients (1,2) and this metastasis is manifested as the progressive aggravation of anemia in the short term, thrombocytopenia and a significantly declined performance status in the majority of patients. Notably, the median survival of patients with metastatic cancer of the bone marrow presenting with thrombocytopenia is only one month and chemotherapy is usually ineffective (3). The presence of micrometastasis in the bone marrow and its effect on prognosis has been shown in patients with identical stages of breast cancer, as defined by tumor size, histological grade, the presence or absence of lymph node metastasis and the expression of hormone receptors (4,5). However, the clinical treatment of such micrometastasis is limited due to the low statistical power of published studies and lack of clinical trials (6). The bone marrow metastasis of breast cancer is common and the current study presents a case of bone marrow metastasis of breast cancer whose peripheral blood cell counts completely returned to within the normal ranges following monotherapy with trastuzumab. This study was approved by the Ethical Committee of the General Hospital of Shenyang Military Region (Shenyang, China) and the patient provided written informed consent.

## Case report

**Figure 1:**
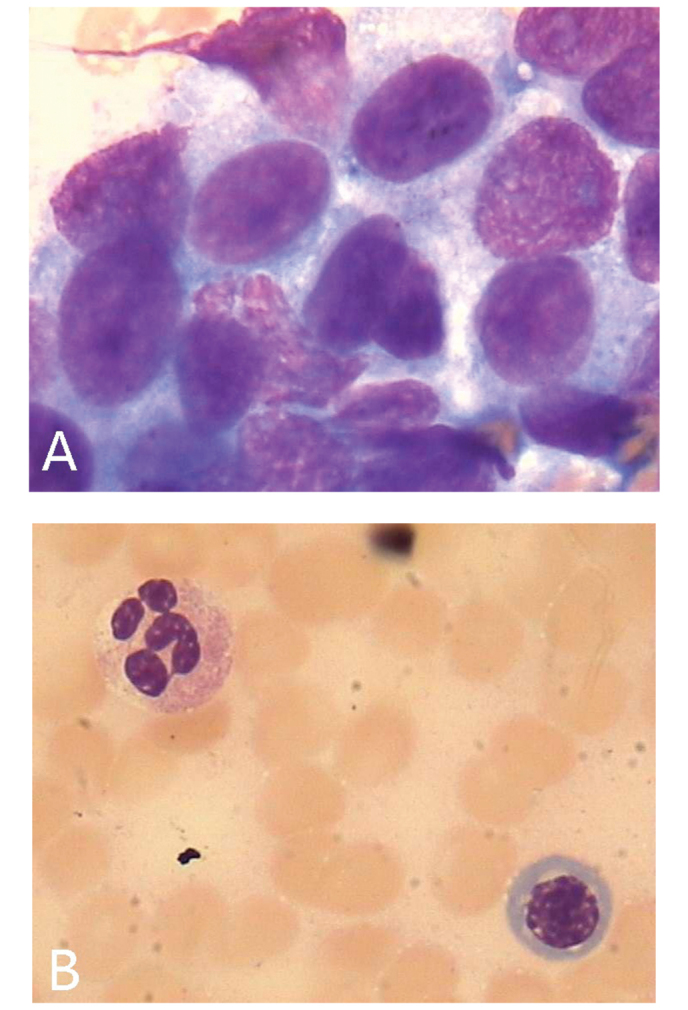
Bone marrow smear tests (A) prior to treatment revealed the presence of metastatic cancer cells and (B) following treatment showed the disappearance of the cancer cells (stain, Giemsa; magnification, ×1,000).

**Figure 2:**
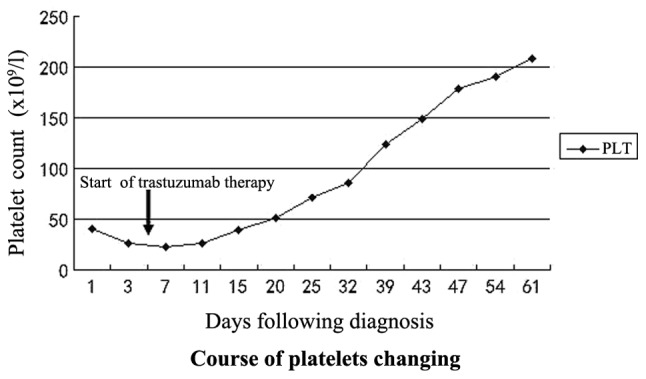
Blood PLT count declined sharply in the early stage of disease, but showed a steady increase following trastuzumab treatment. PLT, platelet.

The current study presents the case of a 41-year-old female who underwent modified radical mastectomy due to left breast infiltrating ductal cancer (pathological stage, IIA; pT1aN1M0) on May 30, 2010 at the General Hospital of Shenyang Military Region (Shenyang, China). The patient was negative for estrogen and progesterone receptors. In addition, the human epidermal growth factor 2 (HER-2) immunohistochemical analysis showed ++/+++ staining and fluorescence *in situ* hybridization (FISH) indicated HER-2 gene amplification. Following the surgery, six cycles of adjuvant chemotherapy (docetaxel plus epirubicin regimen) in combination with sequential adjuvant radiotherapy were conducted. In May 2011, the patient felt pain at multiple sites on the body and subsequent positron emission tomography/computed tomography (CT) examination indicated the following: Left cervical, left supraclavicular, mediastinal, retroperitoneal and pelvic lymph node metastases; superior lobe metastasis of the left lung; peritoneal metastasis; multiple osseous metastases; and tumors in the right appendix region. The patient had an Eastern Cooperative Oncology Group (ECOG) performance status score of 3 and numeric rating scale (NRS) pain score of 8. The results of the routine blood test showed that the lowest peripheral blood cell counts were 1.6×10^9^ cells/l for white blood cells (WBCs), 54 g/l for hemoglobin (Hb) and 26×10^9^ cells/l for platelets (PLTs). In addition, a bone marrow smear test revealed the presence of metastatic cancer cells (Fig. 1A). Trastuzumab monotherapy was subsequently initiated on June 1, 2011, at a loading dose of 8 mg/kg and a maintenance dose of 6 mg/kg every three weeks. During the night following administration of the initial dose of trastuzumab, the patient experienced fever (body temperature of 39.0°C) and more severe pain, which were alleviated by symptomatic treatment. Following trastuzumab treatment, the patient’s PLT count markedly increased (Fig. 2), while the WBC count and Hb concentration increased gradually. Furthermore, following four doses of trastuzumab treatment, the patient’s performance status score (ECOG) decreased to 1 and pain score (NRS) decreased to 2. On August 17, 2011, taxol chemotherapy was initiated at a dose of 75 mg/m^2^ on days two and nine, and was administered on the basis of trastuzumab. In total, seven cycles, each lasting 21 days, were completed. No cancer cells were identified in the subsequent bone marrow smear test (Fig. 1B). CT demonstrated metastatic foci in the left lung, however, the enlarged lymph nodes had subsided, and the tumor in the right appendix region had decreased in size by 50%. In April 2012, the trastuzumab treatment was withdrawn due to brain metastasis and in May 2012, the patient succumbed to disease progression.

## Discussion

Bone marrow micrometastasis has already developed in almost one-third of patients with breast cancer at presentation. Furthermore, large tumor sizes, poor differentiation, lymph node metastasis and negative hormone receptors are risk factors for bone marrow metastasis (1). Severe bone marrow metastasis leads to severe bone marrow suppression, which restricts the efficacy of cytotoxic drugs in such patients. Few individual cases of bone marrow metastasis of breast cancer have been reported (7–10) and the clinical use of cytotoxic drugs continue to pose considerable risks.

Trastuzumab is a humanized monoclonal antibody which is directed against the extracellular domain of the HER-2 gene (11). Combined chemotherapy with trastuzumab has become a standard treatment for HER-2-overexpressing breast cancer (12–14). Despite the lack of high-level medical evidence supporting trastuzumab monotherapy as a treatment for metastatic breast cancer, significant clinical benefits have been highlighted in a phase II clinical study in the treatment of advanced breast cancer with HER-2 gene immunohistochemical scores of >3 or positive FISH tests (15). Furthermore, Tsutani *et al* (16) reported one case of complete remission in a patient with lung metastasis of breast cancer treated with trastuzumab alone. The individual in the present case was a patient with HER-2-overexpressing breast cancer, who developed bone marrow metastasis complicated by severe bone marrow suppression one year following surgery. Such patients are not sensitive to endocrine therapy and chemotherapy poses great risks due to poor performance status and severe bone marrow suppression. The tentative administration of trastuzumab monotherapy in the present case was found to improve the patient’s disease condition gradually and provided the patient with the opportunity to accept combined chemotherapy, which was likely to significantly prolong survival and improve the patient’s quality of life.

Trastuzumab treatment is selected on the premise of HER-2 gene amplification or overexpression. In the current case, immunohistochemical or FISH tests were impractical due to the small quantity of malignant cells identified in the bone marrow smears. Therefore, it was not possible to directly evaluate the HER-2 gene amplification in the bone marrow metastatic foci. This is a fairly common problem in clinical practice, however, in this circumstance, the trial use of trastuzumab was essentially the only available treatment with the prospect of effectively controlling the tumor progression. We considered an evaluation of the rationality of trastuzumab treatment to be essential prior to administration. A meta-analysis showed that the HER-2 gene inconsistency rate between the metastatic and primary foci of breast cancer was ~5.54% (17). According to this result, it is reasonable to guide treatment based on the HER-2 test results of primary foci when the HER-2 gene amplification of the metastatic foci cannot be obtained.

In conclusion, when sufficient clinical evidence for HER-2 gene amplification is available, trastuzumab may be considered as a beneficial treatment option for breast cancer patients with metastases in whom chemotherapy and endocrine therapy are not suitable.
